# No additional stress of sublethal gas supersaturation in a landlocked population of Atlantic salmon (*Salmo salar*) exposed to environmental acidification

**DOI:** 10.1038/s41598-024-53637-5

**Published:** 2024-02-12

**Authors:** Erik Höglund, Lifen Zhou Loland, Rolf Høgberget, Peter Vilhelm Skov, Gaute Velle

**Affiliations:** 1 Norwegian Institute for Water Research, Økernveien 94, 0579 Oslo, Norway; 2 Center of Coastal Research, University of Agder, 4604 Kristiansand, Norway; 3 DTU Aqua, Section for Aquaculture, The North Sea Research Centre, Technical University of Denmark, Hirtshals, Denmark; 4 NORCE Norwegian Research Centre, LFI, Bergen, Norway; 5 Department of Biological Sciences, University of Bergen, Bergen, Norway

**Keywords:** Environmental impact, Freshwater ecology

## Abstract

The landlocked Atlantic salmon population “bleke” faces extinction due to environmental acidification (EA) and hydropower expansion in the Norwegian river Otra. Despite of restoration, unexpected mortality has been reported for this population, possibly due to gas bubble trauma (GBT) from gas supersaturation (GSS) downstream of hydroelectric plants, or EA induced aluminum toxicity. In this study, we applied the allostasis concept to investigate interactions between EA and GBT. This concept comprises additive effects of stressors, which can lead to allostatic overload. Stress coping mechanisms become maladaptive in such situations, which can be indicated by an inability to mount a proper cortisol response in fish. Fish were exposed to sublethal levels of simulated EA (SEA), GSS (a total gas pressure; TGP; of 110%) or a combination of these stressors for six days. Effects on allostatic load were subsequently investigated by assessing the cortisol response to an acute stress test. SEA increased cortisol responsiveness and GSS induced clinical signs of GBT, but no interacting effects between GSS and SEA were observed. This suggests that that 110% TGP did not have an additive effect on the allostatic load imposed by SEA.

## Introduction

Anthropogenic changes, such as habitat fragmentation and destruction, overexploitation and contaminant overload are stressors that negatively affect wild populations (reviewed by Dickens^1^). In accordance with this, there is an increasing number of studies that apply the concept of allostasis to elucidate how anthropogenic stressors affect animals^2–5^. Allostasis was coined by McEwen and Stellar^6^ and comprises the idea that stressors can have additive effects. Moreover, it includes allostatic load, referring to the physiological costs of coping with changes by regaining homeostasis, and allostatic overload, a situation when the costs of adaptation exceed those of stress resilience^7^. In the latter case, the strain imposed on the adaptive responses may alter stress coping ability, leading to maladaptive responses to stress^6,8^. Accordingly, allostatic overload has been associated with decreased fitness^4,9^. In light of this, responses to controlled acute stressors have recently been used to indicate allostatic overload and negative impacts of anthropogenic changes^3,4^.

By modulating the brain and other bodily coping systems, the glucocorticoids play central roles in allostatic processes^10^. In addition to its role in adapting to slow changes, such as circadian changes, glucocorticoids make energy available by stimulating glycogenolysis and suppressing maintenance functions of the body in response to acute stress^11^. Cortisol is the dominant glucocorticoid in teleost fish, and its response to a stressor must be finetuned to ensure an appropriate response. Following the concepts of allostasis and allostatic (over) load, there are studies in teleost fishes showing that chronic or repeated stress results in inability to mount a proper stress response, including suppressed cortisol responsiveness^12,13^. In line with this recent studies show that long-term exposure to environmental acidification (EA) blunts the cortisol response to an acute stressor in Atlantic Salmon (salmon; *Salmo salar*) originating from a unique Norwegian landlocked population^4^.

This landlocked salmon population, commonly referred to as “bleke” (*S. salar* ssp.), inhabits the watershed of the river Otra in the southern part of Norway. It faced near extinction due to a combination of EA and hydropower expansion^14^. Lately, the size of this population has been increasing under a restoration program, including improved habitat quality achieved by an increase in water pH and making spawning areas accessible. Despite these efforts, mortality has been observed in bleke exposed to ambient Otra water^15^. Relatively low levels of EA made the authors suggest that additional stressors might underlie this unexpected high mortality.

A potential stressor is gas supersaturation (GSS). This can occur downstream of hydroelectric powerplants when air is entrained into the penstock system of power plants^16–18^ and when air is entrained in plunge pools below dams^14,19,20^. Gas bubbles can form in tissue and the blood of animals exposed to GSS, inducing gas bubble trauma (GBT), including a variety of lethal and sublethal effects, as reviewed by Pleizier et al.^21^. Accordingly, there are governmental guidelines for total dissolved gas (TGP). In Canada, the limits vary from TGP-103% to 115%, depending on water depth^22^, while the limit is 110% in USA with each state having exceptions to the guidelines with its own regulations^23^. In Norway and elsewhere, there are currently no guidelines or regulations for GSS, even though one national committee in the 1980s concluded that TGP above 110% is likely harmful for fish^24,25^. Still, results from a recent study investigating the impact GSS downstream of hydropower plants found high mortality in bleke at GSS levels of < 110%^26^. Additive effects of other stressors were suggested as an underlying factor for this apparently high impact of GSS^27^.

In this study, we applied the concept of allostatic load to investigate potential interaction effects between the two anthropogenic stressors EA and GSS in bleke. This was done by exposing fish to either GSS similar as those in the study performed by Stenberg et al.^26^ (TGP ≤ 110%), simulated environmental acidification (SEA; pH 5.5 and increased [Al]^4^) at a level just under what has been demonstrated to suppress cortisol responsiveness^4^ or a combination of these potential stressors. After six days of exposure, effects on cortisol responsiveness were investigated by exposing the fish to a standardized acute stress test. We hypothesized that the added allostatic load by exposure to GSS would result in allostatic overload, indicated by a blunted cortisol response in the fish exposed to both GSS and EAS. In addition, potential interaction effects of GSS and AES were investigated by clinical signs of GBT.

## Materiel and methods

### Experimental animals

The fish used in the experiment were 1 + year Atlantic salmon originating from the bleke land-locked population in Otra. They were hatched and reared at the Syrtveit hatchery and weighed 54 ± 11 g (mean ± standard deviation) at the time of experimentation. All fish were kept in water from the Otra River, with pH adjusted to 6.5 and at a natural water temperature. The experiment was performed in September with a water temperature range of 9–10 °C. The fish were kept in tanks (2 × 2 × 1 m; length, width and height) in a flow through system. The oxygen saturation varied between 85 and 100% and fish were fed at a minimal rate (approximately 0.1% of body weight day^−1^) with Skretting 3 mm pellets before the experiment and remained unfed during the experiment. Moreover, fish were kept under continuous dimmed lightning by covering half of the rearing tanks before and during experimentation.

### Experimental protocol

To allow acclimation, 20 fish were inserted in each of eight exposure tanks (1 × 1 × 1 m; length, width and height) with a water depth of 0.3 m for 1 week before exposure. After acclimation, fish were exposed to the following treatments in duplicates for 6 days:

1. Control: Hatchery Otra river water (pH 6.5)
2. Simulated environmental acidification (SEA): Otra river water with pH 5.5 and a nominal Al added concentration of 65 µg l^−1^
3. Gas supersaturation (GSS): Otra river water with a nominal total gas pressure (TGP) of 110%
4. GSS + SEA: Otra river water with a nominal TGP of 110%, and pH 5.5 and a nominal added Al concentration of 65 µg l^−1^.

The added Al concentration of 65 ug l^−1^ In the SEA treatment was chosen based on the study performed by Höglund et al.^4^ demonstrating that reduced stress coping ability became evident in hatchery Otra river water supplemented with Al at a range between 35 and 70 μg l^−1^. Hatchery water was delivered through a blending tube (l = 1.6 m, d = 3.2 cm) to a distribution tank in the SEA, GSS and GSS + SEA treatments. The blending tube had feeding holes for adding acidified water and aluminum at the starting end of the tube. H_2_SO_4_ acidified water was applied to set pH to 5.5, which was controlled by a pH sensor (Hamilton Polylite 120 +) in the distribution tank and proportional integration (PI) regulation of a peristatic pump (Watson-Marlow 300). Diluted aluminum was delivered by a peristaltic pump (Watson Marlow 300) to obtain the nominal 65 µg l^−1^ added Al. The water in the distribution tanks was then distributed to two SEA exposure tanks.

The GSS level was chosen because sublethal effects appear at TGP ≈ 110%^27,28^ and because this is a relevant level of GSS that fish are exposed to downstream from hydroelectric power plants^26^. The GSS water was supersaturated using a modification of the method described in Skov et al.^29^. In brief, water was delivered to an oxygen cone (150 × 55 cm height and width, volume ca. 200L) using a multistage centrifugal pump (Model CRN1S-10 A-P-A-E-HQQE, Grundfos, Bjerringbro, Denmark) and air was added from an oil-free compressor (Luna, ACD1.5-240L). The TGP of water leaving the conical tank was 135%. Then, the TGP was regulated by blending the outflow from the conical tank with regular hatchery water into a 50 L mixing tank. The flow of the regular hatchery water was set by a peristaltic pump (Watson-Marlow 700). During the first two days of the experiment, the peristaltic pump was regulated by a proportional-integral controller (PI) via a TGP sensor (Pont Four, SN111102), resulting in TGP-118% ± 1 in the mixing tank and corresponding to 110% in the exposure tanks. Due to problems with PI regulation, this peristaltic pump was set to a fixed speed of 114 rpm during the last three days of the experiment, which provided a stable TGP level in the mixing tank. The mixing tank had two outflows (7.5 l min^−1^) to two 50 l distribution tanks. One of the distribution tanks delivered water to two exposure tanks directly and resulted in a TGP level of 110% GSS exposure. The outflow from the second distribution tank was connected to a blending tube for delivery of acidified water and Al (see above for description) to a second distribution tank. As in the SEA treatment, H_2_SO_4_ acidified water was applied to set pH 5.5, and was controlled by a pH sensor (Hamilton Polylite 120+) and PI regulation of a peristatic pump (Watson-Marlow 300). Diluted aluminum was delivered by a peristaltic pump (WatsonMarlow 300) to obtain a nominal concentration of 65 µg [Al] l^−1^. After mixing in the distribution tank, the treated water was distributed to two GSS + SEA exposure tanks.

All distribution tanks had an outflow of 3.15 l min^−1^ that discharged into the exposure tanks with an overflow.

During the experiment period, TGP in the GSS and SEA + GSS exposure tanks were measured manually twice daily with a gas supersaturation meter (Clark Fork, model SM1, serial 0078). TGP in the SEA and control treatment was checked twice at day one, two, four and five. The TGP in the GSS and SEA + GSS treatments were 111 ± 0.6% and 111 ± 0.7% (mean ± S.D.), respectively. Due to restricted inflow, TGP was 90% during the first day in one of the control tanks. TGP in the control and SEA treatment varied between 99.6 and 100.6% during the remaining experiment. Total aluminum [Al_tot_] was analyzed in two water samples withdrawn in the beginning of the treatment (day 1 or 2) and in the end of the treatment (day 5 or 6) from each tank. Water samples were frozen and stored at − 20 °C before the [Al_tot_] analysis.

To investigate the effect of treatment on cortisol responsiveness, five fish from each exposure tank were exposed to a standardized acute stress test at day six of the exposure period. This was done by transferring fish to 1.5 × 0.5 × 0.2 m (length × width × depth) chambers where single fish were kept with the water surface just above the dorsal fin for 30 min. This acute stress test has been used to detect both heritable and environmental effects in stress coping ability in teleost fishes^3,4,30–33^. At the end of the stress test, fish were netted and euthanized in an overdose of MS 222 (500 mg l^−1^) and 1ml blood was collected from the caudal vasculature of fish using a syringe pretreated with heparin. Thereafter, blood samples were rapidly transferred to Eppendorf tubes and centrifuged at 1500*g* for 10 min. Following centrifugation, the blood plasma samples were frozen on dry ice and stored at − 80 °C. This procedure resulted in samples sizes of n = 10 for all four treatments. For determination of Al in the gills, two fish from each tank (n = 4 per treatment) were killed by a blow to the head and the second gill on the right side were dissected out, snap-frozen on dry ice, and stored at − 80 °C. In addition, five fish from each tank (n = 10 per treatment) were euthanized and investigated for clinical sign of gas bubble trauma.

### Analysis Al

Gill tissue was freeze-dried, weighed, and then digested in concentrated, trace metal-grade nitric acid (HNO_3_) overnight at 50 °C. Samples were then diluted in 10% HNO_3 _and trace elements measured by mass-spectrometry (Agilent 7700 Q-ICP-MS). For quality control, we concurrently ran certified reference materials: DORM-4 (fish protein) and DOLT5 (dogfish liver), both from the National Research Council of Canada, and IAEA-436 (tuna Fish flesh homogenate) from the International Atomic Energy Agency. Results are expressed as μg Al per g of gill dry weight. Al in water samples were analyzed using ICP-MS.

### Analysis of plasma cortisol

Cortisol in plasma was analyzed using a commercially available DetectX® cortisol enzyme immunoassay kit (Arbor Assays, Ann Arbor, MI, USA) following the manufacturers protocol. The absorbance of the prepared ELISA plate was read in a plate reader at 450 nm and the concentrations were calculated using the four-parameter logistics curve.

### Clinical signs of GBT

Immediately after sampling, fish were examined for clinical signs of GBT using a dissection microscope at 25 to 50 × magnification. Investigated clinical signs were; bubbles in the lateral line, mouth, fins, the orbital rim around the eyes and gills. In addition, the eyes were examined for exophthalmia (“pop-eye”).

### Statistics

#### Gill Al, and Al_tot_

A Mann–Whitney U test (M–W U tests) were applied to investigate differences in [Al_tot_] and gill Al between treatments with no exposure to SEA (control and SEA) vs. treatments with exposure to SEA (SEA and SEA + GSS). In addition, differences between SEA vs. SEA + GSS and SEA vs. control treatments were investigated with M-W U tests. Bonferroni corrections (α/3) were applied to compensate for multiple comparisons.

Fisher’s exact probability test was applied to analyze for differences in frequency of fish with BT between the GSS and SEA + GSS treated groups. The effects of SEA and GSS on cortisol responsiveness were investigated by two-way analysis of variance (ANOVAs) with the total gas pressure and acidification as independent variables.

### Statements

The study is reported in accordance with ARRIVE guidelines (https://arriveguidelines.org). Furthermore, the experiments was conducted in accordance with the Guidelines of the European Union Council (86/609/EU) and Norwegian legislation for the use of laboratory animals. The experimental protocol was approved by the ethics committee of the Norwegian food safety authority (Permit number 200130).

## Results

### Water and gill Al

SEA treatment of the hatchery water resulted a general increase in [Al_tot_] (M–W U test; Z = 3.3, P < 0.001). Furthermore, [Al_tot_] in the control and the GSS treatment water did not differ significantly (P < 0.65). Neither did the GSS and SEA + GSS treatments differ in [Al_tot_] (P < 0.03; α = 0.016 after Bonferroni correction), Table 1.

**Table 1:** Total Aluminum [Al _tot_] in hatchery river Otra water (Control; pH 6.5 and TGP 100%), gas super saturated hatchery water (GSS; pH 6.5 and a total gas pressure (TGP) of 110%), hatchery water with simulated environmental acidification (SEA; pH 5.5 + Al; and TGP 100%) or the combination of GSS and SEA (GSS + SEA; pH5.5 + Al and TGP 110%), and Gil aluminum in Atlantic salmon (*Salmo salar* ssp. bleke) originating from landlocked population in the River Otra kept in these water treatments for six days. For SEA, H_2_SO_4_ was used to acidify the water and AlCl_3_.6H2O was added to a nominal concentration of 67 μg [Al] l^−1^. group. Values are median (− 25th percentile + 75%th percentile), Significance level P < 0.016 after Bonferroni correction; α/3) and n = 4.

	Control	GSS	M–W U test	SEA	GSS + SEA	M–W U test
[Al_tot_] (ng ml^−1^)	95 (− 95 + 109.5)	95.5 (− 92 + 103)	Z = 0.43; P < 0.65	170 ± (− 170 + 175)	157 (− 150 + 160)	Z = 2.2; P < 0.03
Gill Al (ng g^−1^ dry wieght)	3.0 (− 2.6 + 3.8)	2.7 (− 2.5 + 3.4)	Z = 0.4; P < 0.69	327 (− 240 + 524)	334 (− 295 + 368)	Z = 0.0; P = 1

Furthermore, exposure to SEA (SEA and SEA + GSS) resulted a general increase in Gill Al (M-W U test; Z = 3.3, P < 0.001) compared to fish not excposed to SEA (control and GSS) (M-W U test; Z = 3.3, P < 0.001). However, there were no significant difference in gill Al between control and GSS exposed fish. Neither did gill Al differ between fish exposed to SEA or SEA + GSS significantly (0.4; P < 0.69).

### Clinical signs of GBT

**Figure 1:**
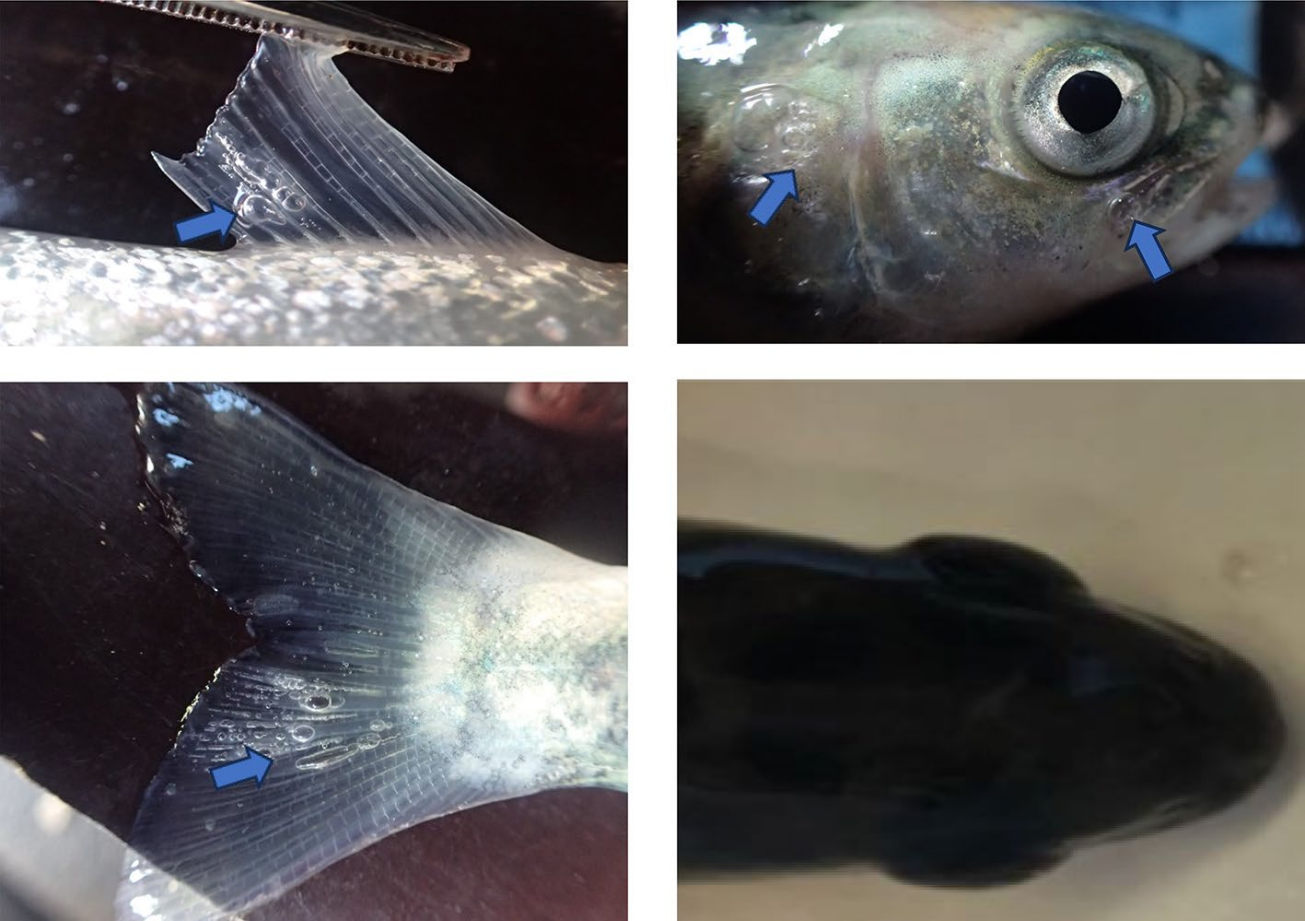
Clinical signs off gas bubble trauma, including bubbles under skin (**A**–**C**; indicated by arrows) and exophthalmos (pop-eyes; **D**), in Atlantic salmon (*Salmo salar* ssp. bleke) originating from landlocked population in the River Otra. Fish were exposed to gas supersaturated (a total gas pressure of 110%) water for 6 days.

Exposure to GSS resulted in clear bubbles in the fins, the orbital rim, in the mouth and/or gills in 9 out of 10 fish Fig 1. Two fish displayed signs of exophthalmia (pop-eye), Fig. 1. In the combined exposure of GSS + SEA, clear gas bubbles around the eyes, in the mouth, gills and/or fins were found in 6 out of 10 fish, in addition light reflexes could not be distinguished from gas bubbles, and thus was categorized as unclear signs of GBT in 2 of the fish examined, Table 2. There was no difference in the frequency of fish with clinical sign of BT between groups of fish exposed to GSS and groups of fish exposed to SEA + GSS (Fishers exact test; P < 0.30).

**Table 2:** Numbers of fish with signs of gas bubble trauma. Atlantic salmon (*Salmo salar ssp. Bleke*) originating from a landlocked population in the Otra river, were exposed to water from river Otra (Control, pH 6.5), or this water with gas supersaturation (GSS; TGP-110%), simulated environmental acidification (SEA; pH 5.5 + Al), and the combine (GSS + SEA; pH 5.5 + Al, TGP-110%), for six days. H_2_SO_4_ was used to acidify water to pH5.5; AlCl_3_.6H_2_O was diluted to obtain a nominal concentration of 67 µg [Al] l^−1^ for SEA. The nominal total gas pressure (TGP) in GSS was 110% in the exposure tanks, while the TGP level was 100% in treatments with no GSS.

	Fish inspected (n)	Gas bubbles	Exophtalmia (pop eye)	Internal hemorrhage	Fish with clinical signs	Fish with uncertain signs
Control	10	0	0	0	0	1
GSS	10	9	2	0	9	0
SEA	10	0	0	0	0	0
GSS + SEA	10	6	0	0	6	2

Fish that had not been exposed to GSS (SEA and Control) displayed no clear signs of gas bubble formation. One control fish was categorized as having unclear signs of GSS.

There was no mortality observed in any of the treatment groups.

### Cortisol responsiveness

**Figure 2:**
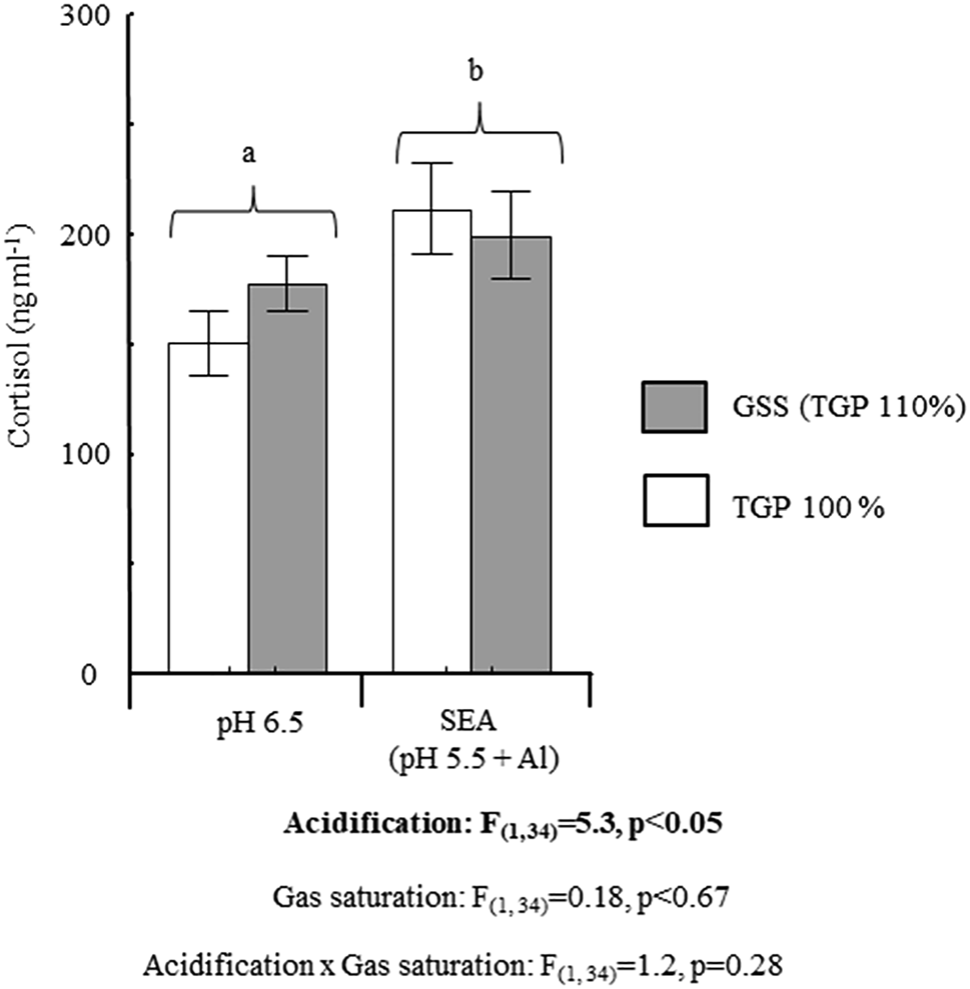
Plasma cortisol levels after an acute stress test in Atlantic salmon (*Salmo salar* ssp. bleke) originating from landlocked population in the River Otra. Fish were exposed to either gas super saturation (GSS; pH 6.5 and a total gas pressure (TGP) of 110%), simulated environmental acidification (SEA; pH 5.5 + Al; and TGP 100%), the combination of GSS and SEA (GSS + SEA; pH5.5 + Al and TGP 110%) or Hatchery river Otra water (Control; pH 6.5 and TGP 110%), for 6 days. For SEA, H2SO4 was used to acidify the water and AlCl_3_⋅6H_2_O was added to a nominal concentration of 67 μg [Al] l^−1^. Values are means ± standard error of mean, different letters indicate significant differences (p < 0.05) and n = 10 in each group.

Fish exposed to simulated acidification (SEA and SEA + GSS) had significantly higher plasma levels of cortisol to the acute stress, compared to fish not exposed to SEA (GSS and control) (ANOVA; F_(1,34)_ = 5.4, P < 0.05), Fig. 2. However, there was no significant effect of GSS (ANOVA; F_(1,34)_ = 0.18). Moreover, there were no significant interaction effect between SEA and GSS (ANOVA; F_(1,34)_ = 1.2; P < 0.28), Fig. 2.

## Discussion

No mortality was observed in the present study at a TGP level of 111% and water depth of 0.3 m, resulting in GSS level of 108% after depth compensation at the bottom where the fish resided. Somewhat in contrast to this, Stenberg et al.^26^ reported significant increased mortality at TGP level of 110% in bleke kept in cages downstream of a hydroelectric powerplant. Furthermore, after compensating for a depth of 0.3–0.55 m they concluded that a mortality occurred at a level of 107–105%. Moreover, in the aforementioned study it was pointed out that this threshold for mortality was ≈ 2% lower than previously reported in pacific salmon^27,28^. Likewise, Krise and Herman^33^ reported acute mortality in Atlantic salmon exposed to a TGP level of 117%, while no mortality was observed at 110%. Stenberg et al.^26^ suggested several factors underlying the discrepancy in mortality between their study and the study performed by Krise and Herman^33^. Among them were physiological differences between the landlocked bleke population and anadromous populations. Our study does not lend support to this. We observed clinical signs of GBT, as observed in the study performed by Stenberg et al.^26^, but no mortality at TGP level of 111%. Additional stressors in field studies compared to laboratory studies may contribute to the higher mortality in the study performed by Stenberg et al.^26^. In addition, it is important to note that the fish were smaller (18.7 g) in the study performed by Stenberg et al.^26^ compared to the fish in the current study (54 g) and it cannot be excluded size that differences contributes to differences in mortality. This is supported by a study performed by Kovak et al.^34^, showing a negative relationship between age and proportion of fish with external symptoms of GBT in white sturgeon (*Acipenser transmontanus*), and unpublished results from our lab suggesting a higher tolerance towards GSS in smaller juvenile stages than in larger juvenile stages in Atlantic salmon.

Continuous pH logging in Otra has shown that heavy rainfall in a hydroelectric powerplant catchment with low buffering capacity can lead to local EA (pH 5.0–5.5) episodes^15^. Following this, there have been several studies investigating the impact of EA on the bleke population^3,4,35^. Detrimental effects of EA are closely linked to aluminum ions [Al^3+^] being mobilized from surrounding soils. These ions then form complexes with water molecules that bind to fish gills at moderately low pH, affecting membrane permeability by inducing mucus production and cell swelling^36^. Lethal effects of environmental acidification have been reported when gill Al exceeds 400 μg g^−1^ dry weight in Atlantic salmon^36^. Moreover, sublethal effects, including effects on cortisol responsiveness, have been reported at lower gill aluminum levels in bleke^3,4^. Furthermore, Höglund et al.^4^ demonstrated a positive relationship between cortisol responsiveness and impact of SEA (i.e., increasing water [Al tot] at pH 5.5) up to a level inducing a gill Al accumulation of 230 μg g^−1^ dry weight. However, above this gill Al level, SEA had a dampening effect on cortisol responsiveness. This has been suggested to reflect an inability to mount a proper cortisol response, indicating allostatic overload^3,4,12^. According to the latter, several studies in have shown that prolonged exposure to aquatic environmental contaminants and other chronic or repeated stressors suppress the cortisol response to an acute stressor in teleost fishes^11,37–39^. In the present study, there was a general increase in stress responsiveness in fish exposed to SEA. However, we could not detect any significant difference in cortisol responsiveness between fish exposed to SEA alone and fish exposed to a combination of SEA and GSS. According to the study performed by Höglund et al.^4^, this suggests that 110% GSS did not increase total stress burden to reach allostatic overload. In this situation, a dampening effect on cortisol responsiveness would be expected in fish exposed to GSS + SEA. In a previous study, unexpected high mortality was observed in bleke kept in cages downstream a hydroelectric powerplant at sublethal levels of SEA (gill Al 173 μg g^−1^ dry weight^15^ leading the authors to suggest that an underlaying factor for this mortality was additional stress induced by GSS. Our study showed no interaction effects between GSS and SEA in mortality or allostatic load, and does not lend support to this. However, as stated by Stenberg et al.^26^., and other factors may contribute to the total stress burden in fishes kept in cages, including being exposed to uncontrollable additional stressors and fluctuating TGP levels.

In conclusion, this study demonstrated increased cortisol responsiveness in fish exposed to SEA and clinical signs of GBT in fish exposed to 111% TGP. An increasing number of rivers with GSS are found downstream of hydroelectric power plants and dams, such as in Norway, China, and North America^18,39,40^. Sub-lethal effects and increased mortality in fish and benthic invertebrates commences from about 105% TGP^21,41^. There are added stressors in addition to GSS in many of these rivers, such as, acidification, eutrophication, impoundments, surface-water abstractions and land-use change^1,14^. We would expect interaction effects from these stressors. However, we observed no interaction effects in mortality or stress responsiveness between GSS and acidification. This suggests that a TGP level, inducing clinical signs of TGP, did not have an additive effect on the allostatic load imposed by environmental acidification. This signifies that regulations for TGP should be based on acute or subacute effects caused by GSS, and not be compensated for additive effects caused by other stressors. However, it is important to stress that fish are exposed to higher levels of TGP and possibly also higher levels of EA in the watershed of the Otra river. Thus, further studies, including higher impact of EA and higher levels of TGP, might reveal additive effect between these anthropogenic stressors in bleke and other fishes. Furthermore, both EA and GSS have been reported to decrease swimming capability^42–45^, suggesting that further studies of interaction effects between TGP and EA should include measures of physiological performance.

## Data Availability

The datasets used and/or analysed during the current study available from the corresponding author on reasonable request.
